# Selective proliferative response of microglia to alternative polarization signals

**DOI:** 10.1186/s12974-017-1011-6

**Published:** 2017-12-04

**Authors:** Giovanna Pepe, Marcella De Maglie, Lucia Minoli, Alessandro Villa, Adriana Maggi, Elisabetta Vegeto

**Affiliations:** 1 Center of Excellence on Neurodegenerative Diseases and Department Pharmacological and Biomolecular Sciences, University of Milan, Via Balzaretti, 9, 20133 Milan, Italy; 2 Mouse and Animal Pathology Laboratory (MAPLab), Fondazione Filarete, Viale Ortles, 22/4, 20139 Milan, Italy; 3 Department of Veterinary Medicine, University of Milan, Via Celoria, 10, 20133 Milan, Italy

**Keywords:** Interleukin-4, Microglia, Proliferation, Estrogen

## Abstract

**Background:**

Microglia are resident myeloid cells of the central nervous system (CNS) that are maintained by self-renewal and actively participate in tissue homeostasis and immune defense. Under the influence of endogenous or pathological signals, microglia undertake biochemical transformations that are schematically classified as the pro-inflammatory M1 phenotype and the alternatively activated M2 state. Dysregulated proliferation of M1-activated microglia has detrimental effects, while an increased number of microglia with the alternative, pro-resolving phenotype might be beneficial in brain pathologies; however, the proliferative response of microglia to M2 signals is not yet known. We thus evaluated the ability of interleukin-4 (IL-4), a typical M2 and proliferative signal for peripheral macrophages, to induce microglia proliferation and compared it with other proliferative and M2 polarizing stimuli for macrophages, namely colony-stimulating factor-1 (CSF-1) and the estrogen hormone, 17β-estradiol (E_2_).

**Methods:**

Recombinant IL-4 was delivered to the brain of adult mice by intracerebroventricular (i.c.v.) injection; whole brain areas or ex vivo-sorted microglia were analyzed by real-time PCR for assessing the mRNA levels of genes related with cell proliferation (*Ki67*, *CDK-1*, and *CcnB2*) and M2 polarization (*Arg1*, *Fizz1*, *Ym-1*) or by FACS analyses of in vivo BrdU incorporation in microglia. Primary cultures of microglia and astrocytes were also tested for proliferative effects.

**Results:**

Our results show that IL-4 only slightly modified the expression of cell cycle-related genes in some brain areas but not in microglia, where it strongly enhanced M2 gene expression; on the contrary, brain delivery of CSF-1 triggered proliferation as well as M2 polarization of microglia both in vivo and in vitro. Similar to IL-4, the systemic E_2_ administration failed to induce microglia proliferation while it increased M2 gene expression.

**Conclusions:**

Our data show that, in contrast to the wider responsiveness of peripheral macrophages, microglia proliferation is stimulated by selected M2 polarizing stimuli suggesting a role for the local microenvironment and developmental origin of tissue macrophages in regulating self-renewal following alternative activating stimuli.

**Electronic supplementary material:**

The online version of this article (10.1186/s12974-017-1011-6) contains supplementary material, which is available to authorized users.

## Background

Microglia are the resident population of innate immune cells of the central nervous system (CNS), representing up to 10% of total brain cells, with differences in cell density depending on the brain area [1]. It has been demonstrated that microglia derive from yolk sac progenitors that colonize the neuroepithelium in early stages of embryogenesis [2] and are maintained by self-renewal from embryonal to adult life by constant and rapid cell turnover, without the contribution of circulating monocytes [3–5].

Under physiological conditions, microglia display a ramified shape and actively sustain neuronal activity. In analogy to peripheral tissue macrophages, micro-environmental and pathogenic stimuli activate microglia to acquire different immunometabolic properties that are schematically defined as classical, or “M1,” activation phenotype that is induced by bacterial or immune molecules, such as lipopolysaccharide (LPS) or TNFα, and promotes an inflammatory cytotoxic response; an alternative, “M2” phenotype that is activated in response to stimuli, such as interleukin (IL)-4 and IL-13, and allows tissue repair and resolution of inflammation [6]. Although M1- and M2-like activation phenotypes have been described in chronic brain diseases, still debated are their specific mediators and functions during neurotoxicity.

Recently, it has been hypothesized that dysregulated proliferation of microglia plays a detrimental role on neuronal cell viability, as shown in the case of experimental models of neurodegenerative disorders, such as Alzheimer’s disease [7–9], amyotrophic lateral sclerosis [10, 11], and Huntington’s disease [12], as well as acute CNS injuries [13, 14]. M1-activating stimuli were demonstrated to induce microglia proliferation [15, 16], while the ability of microglia to proliferate in response to M2 signals has not been addressed yet. On the contrary, IL-4 has been recently shown to induce peripheral macrophage proliferation [17]; moreover, brain IL-4 levels may increase during pathological events, while administration of this molecule associates with beneficial effects on neuronal survival [18–21]; thus, promoting proliferation of microglia endowed with pro-resolving properties may have beneficial effects on disease progression and offer new therapeutic strategies to selectively modulate disease outcome. Interestingly, also endocrine signals such as the sex steroid hormone 17β-estradiol (E_2_) were shown to modulate the phenotypic activation and to induce proliferation of peritoneal macrophages in vivo, exerting anti-inflammatory effects also in diverse macrophage populations including microglia [22, 23].

The aim of this study was thus to assess whether the M2-like polarization effects of IL-4 in microglia are associated with a proliferative response. To this purpose, we analyzed cell proliferation in the mouse brain following the intracerebroventricular (i.c.v.) injection of IL-4, an in vivo method that efficiently induces alternative activation of microglia [24]. Our data show that, differently from peripheral macrophages, microglia do not proliferate in response to IL-4; similarly, E_2_ polarizing effects in microglia are not associated with cell proliferation, which is instead induced by colony-stimulating factor-1 (CSF-1), a typical macrophage growth factor that also induces M2 polarization effects.

## Methods

### Animals and treatments

C57BL/6 female mice of 4 months of age were supplied by Charles River Laboratories (Calco, Italy). Animals were allowed to food and water access ad libitum and kept in temperature-controlled facilities on a 12-h light and dark cycle. Animals were housed in the animal care facility of the Department of Pharmacological and Biomolecular Sciences at the University of Milan.

Animal investigation has been conducted in accordance with the ethical standards and according to the Declaration of Helsinki and according to the Guide for the Care and Use of Laboratory Animals, as adopted and promulgated by the US National Institute of Health, and in accordance with the European Guidelines for Animal Care and Use of Experimental Animals.

Female mice identified in metaestrous phase were selected for our analyses; the phase of the reproductive cycle in female mice was assessed by blind analysis of vaginal smears mounted on glass microscope slides and stained with May-Grünwald-Giemsa method (MGG Quick Stain Kit; Bio-Optica, Milan, Italy) according to the manufacturer’s protocol.

Mice were injected i.p. with 100 μl of 0.9% NaCl containing 5 μg of mouse recombinant IL-4 (Peprotech, London, UK). 17β-estradiol (E_2_; Sigma-Aldrich Corp., Milan, Italy) was administered by a 100-μL subcutaneous (s.c.) injection of 5 μg/kg E_2_ dissolved in corn oil by stirring in the dark and at room temperature o/n.

For in vivo BrdU labeling experiments, mice were injected i.p. with 30 μl of a 10 mg/ml solution of BrdU (Sigma-Aldrich) dissolved in 0.9% NaCl. Animals were sacrificed 2 h after BrdU injection (*n* = 6).

Two-day-old newborn rats (CD rats, Charles River) were supplied by Charles River Laboratories for the preparation of microglia and astrocytes primary cultures.

### Intracerebroventricular injection

Intracerebroventricular (i.c.v.) injections were performed as previously described [24]. Briefly, mice were anesthetized with a s.c. injection of a ketamine/xylazine solution (78 and 6 mg/kg, respectively) and positioned on a specific stand for surgical operation. Injections in the third cerebral ventricle were performed according to specific stereotaxic coordinates (bregma, − 0.25 mm; lateral, 1 mm; depth, 2.25 mm). IL-4 was injected at the concentration of 5 μg in 3.0 μl of 0.9% NaCl for expression studies on purified adult microglia or 250 ng in 2.5 μl 0.9% NaCl for all other experiments; CSF-1 (Peprotech) was injected at the concentration of 1 μg in 3 μl of 0.9% NaCl. Animals injected with the same volume of vehicle alone (0.9% NaCl) were used as controls. Injections were made using a 26S–gauge Hamilton syringe at a rate of 0.1 μl/3 s, and the needle was kept in place for additional 30 s and then removed slowly. The skin incision was closed with a suture and animals were allowed to recover for 24 h before sacrifice by a lethal ketamine/xylazine solution (150 and 12 mg/kg, respectively). Whole brains were removed and immediately processed for microglia isolation; brain areas (frontal cortex, hippocampus, and striatum of left hemisphere, ipsilateral to the injection site) were collected, immediately frozen on dry ice, and stored at − 80 °C until processed for RNA preparation. Whole brains were fixed in 4% formalin solution and stored until processed for immunohistochemistry.

### Isolation of peritoneal macrophages

After 24 h of IL-4 or vehicle i.p. injections, peritoneal cells were isolated by peritoneal lavage and incubated with anti-CD11b antibody-loaded MicroBeads (Miltenyi Biotec, Bologna, Italy), as previously described [22]. Briefly, 5 ml of pre-chilled 0.9% NaCl were injected into the peritoneal cavity using a 21 G needle; cell suspension was recovered and centrifuged; following incubation with ammonium-chloride-potassium (ACK) solution (0.15 M NH4Cl, 1 mM KHCO3, 0.1 mM EDTA; pH 7.3) for 5 min at 4 °C, cells were either plated and treated as described in the primary cell culture section or incubated with cd11b beads for macrophage purification. For cd11b+ cell isolation, after washing with PBS + 0.5% BSA, cells were resuspended in 90 μL PBS + 0.5% BSA and incubated with 10 μL CD11b MicroBeads for 15 min at 4 °C. After washing, cells were resuspended in 500 uL PBS + 0.5% BSA and applied to MS Miltenyi columns (Miltenyi Biotec) for the magnetic separation procedure. After three washing steps, CD11b-positive cells were eluted from the columns and counted. CD11b-positive cells obtained from each animal were divided into three aliquots; two aliquots of 2.5 × 10^5^ cells each were used to detect BrdU and Ki67 by flow cytometry, the remaining aliquot was stored in TRIzol reagent (Invitrogen-Thermo Fisher Scientific, Milan, Italy) and used for gene expression studies.

### Isolation of microglia from adult brains

After i.c.v. treatments, microglia cells were sorted from adult brains (*n* = 4), as previously described [24]. Briefly, whole brains were dissected and washed in Hank’s balanced salt solution (HBSS; Life Technologies-Thermo Fisher Scientific); after removing the meninges, enzymatic cell dissociation was performed using Neural Tissue Dissociation Kit P (Miltenyi Biotec), with some protocol modifications: after enzymatic digestion with papain, samples were dissociated mechanically, homogenized, and filtered through a 40-μm cell strainer. After extensive washes in HBSS, myelin was removed by suspending samples in 10 ml of cold 0.9 M sucrose solution and centrifuging the dissociated brain cells at 850*g* and 4 °C for 10 min without braking. Floating myelin and the supernatant were discarded, and cells were processed for microglia magnetic sorting by incubating with CD11b MicroBeads (diluted 1:10 in PBS + 0.5% BSA; Miltenyi Biotec) for 15 min at 4 °C; after washing, cells were suspended in 500 μl of PBS + 0.5% BSA and applied to a magnetic column to purify CD11b+ cells. Immediately after isolation, cells were stored in TRIzol reagent (Invitrogen-Thermo Fisher Scientific) for gene expression.

### Primary cell cultures

#### Peritoneal macrophages

For in vitro assay, peritoneal cells were incubated with ACK solution, as described above, counted, and seeded at the concentration of 1 × 10^6^ cells/ml in RPMI + GlutaMax (Gibco™-Thermo Fisher Scientific) supplemented with 10% endotoxin-free FBS, 1% penicillin/streptomycin, and 1% Na pyruvate (RPMI + 10% FBS). After 45 min and several washes in PBS, medium was replaced with RPMI + 10% FBS for IL-4 and CSF-1 treatment and in RPMI w/o phenol red supplemented with 10% dextran-coated charcoal (DCC)-FBS (RPMI + 10% DCC) for E2 treatment. After 3 h, cells were treated for 16 h with vehicle or 20 ng/ml of recombinant murine IL-4 or 20 ng/ml of recombinant murine CSF-1. For estrogen treatment, cells were treated on the next day for 3 h with vehicle (0.01% ethanol (EtOH)) or E_2_ 10^5^ M.

#### Astrocytes and microglia cell cultures

Primary cultures of glial cells were prepared from 2-day-old newborn rats as previously described [25]. After meninges removal, brains were mechanically dissociated and digested in a solution of 2.5% trypsin (Sigma-Aldrich) and 1% DNAse (Sigma-Aldrich), filtered through a 100-μm cell strainer, and seeded at the confluence of 5 × 10^6^ in a 75-cm^2^ flask in minimum essential Eagle’s medium (MEM) supplemented with 10% FBS, 0.6% glucose, 1% penicillin and streptomycin, and 1% L-glutammine (MEM + 10% FBS). Glial cells were grown at 37 °C under a humidified 5% CO_2_ and 95% air atmosphere, and medium was replaced every 3 days. After 10 days, microglia were obtained by shaking the confluent monolayer of mixed glial cells at 260 rpm for 2 h and seeded in 12-well plates at the confluence of 5 × 10^5^ cells/well. The medium was changed with MEM + 15% FBS or MEM + 5% FBS 30 min after microglia plating in order to remove contaminating cells. In order to purify astrocytes, enriched astroglia cultures following microglia separation were incubated with 5 mM L-leucine methyl ester (Sigma-Aldrich) to eliminate contaminating microglia cells and seeded in six-well plates at the confluence of 5 × 10^5^ cells/well in MEM + 15% FBS or MEM + 5% FBS.

Astrocytes and microglia were treated for 16 h with 20 ng/ml of recombinant rat IL-4, 20 ng/ml of recombinant rat CSF-1 or vehicle. For in vitro proliferation assay, cells were treated with 10 μM BrdU for 2 h before cell processing for flow cytometry analysis.

### Flow cytometry analysis

For Ki67 staining, cells were fixed in 4% paraformaldehyde for 15 min, extensively washed with 125 mM glycine in PBS and permeabilized o/n in PBS containing 0.5% Triton X-100 and 1% BSA, at 4 °C. Cells were incubated with rabbit anti-mouse Ki67 antibody conjugated with eFluor660 (Affymetrix eBioscience, Milan, Italy) diluted 1:100 in incubation solution (PBS containing 0.5% Triton X-100 and 0.05% BSA) at room temperature for 1 h. After extensive washes in PBS, cells were analyzed with a flow cytometry system (NovoCyte® 3000 flow cytometer, ACEA Biosciences, San Diego, CA) and analyzed with NovoExpress® Software (ACEA Biosciences).

For BrdU staining, ex vivo peritoneal cells or in vitro primary cells, detached by 0.25% Trypsin-EDTA (Life Technology) for astrocytes or Accutase (Merck-Millipore, Vimodrone (MI), Italy) for microglia, were fixed and permeabilized in 70% EtOH for 30 min at 4 °C and DNA was denaturated with 2 N HCl/0.5% Triton X-100 and incubated 30 min at room temperature. Cells were washed with 0.1 M sodium tetraborate (pH 8.5) and incubated with rat anti BrdU antibody (AbD Serotec—Bio-Rad, Segrate, Italy) diluted 1:100 in incubation solution (PBS containing 0.05% Tween-20 and 1% BSA). After washes in PBS + 1% BSA, cells were incubated with Alexa647-conjugated goat anti-rat secondary antibody (1:200 in incubation solution; Molecular Probes, Monza, Italy) for 1 h at room temperature. After extensively washing with PBS, ex vivo peritoneal macrophages were resuspended in PI solution (H2O containing 10% NP40, 1 mg/ml RNase A and 5 μg/ml PI stock; Sigma-Aldrich), instead primary cells were resuspended in PBS. Samples were analyzed using NovoCyte® 3000 flow cytometer and analyzed with NovoExpress® Software (ACEA Biosciences). Animals with no pulse of BrdU and in vitro samples without BrdU treatment were used for gating strategy to evaluate non-specific signals. Doublets were removed based on FL2 scatter width (FL2-W)/FL2 scatter area (FL2-A).

### RNA preparation and expression analyses

Following 24 h of vehicle or IL-4 (250 ng) i.c.v. treatment, brain area (frontal cortex, striatum, and hippocampus) were first homogenized using steel beads and tissue Lyser (Qiagen, Milan, Italy) at 28 Hz, for three cycles of 20 s followed by 30 s, on ice and in RLT buffer. Total RNA from tissue or cells was purified using RNeasy minikit protocol (Qiagen), according to the manufacturer’s instructions, including a step with deoxyribonuclease incubation. For real-time PCR, 1 μg RNA (500 ng for striatum and primary cultures, 100 ng for isolated microglia, 300 ng for ex vivo peritoneal macrophages) was used for cDNA preparation using 8 U/μl of Moloney murine leukemia virus reverse transcriptase (Promega, Milan, Italy) in a final volume of 25 μl; the reaction was performed at 37 °C for 1 h, and the enzyme inactivated at 75 °C for 5 min. Control reactions without the addition of the reverse transcription enzyme were performed (data not shown). A 1:4 cDNA dilution was amplified using GoTaq®qPCR Master Mix technology (Promega) according to the manufacturer’s protocol. The PCR was carried out in triplicate on a 96-well plate using QuantStudio® 3 real-time PCR system (Applied Biosystems-Thermo Fisher Scientific) with the following thermal profile: 2 min at 95 °C; 40 cycles, 15 s at 95 °C, 1 min at 60 °C. Primer sequences are reported in Additional file 1: Table S1. Data were analyzed using the 2^−ΔΔCt^ method.

### Immunohistochemistry

Brains were trimmed using a brain matrix (Adult Mouse Brain Slicer Matrix BSMAS005-1, Zivic Instruments, Pittsburgh, PA, USA), and sections were routinely processed, paraffin embedded, and sectioned in 4-μm serial sections. After heat-induced epitope retrieval, performed in Dewax and HIER Buffer H (TA-100-DHBH, Thermofisher Scientific, Waltham, MA, USA) for 40 min at 94 °C, sections were incubated with a 10% normal goat serum for non-specific binding blocking. Sections were immunostained with rabbit polyclonal anti-Ki67 antibody (Clone SP6, Thermo Fisher Scientific, Waltham, MA, USA), incubated with biotinylated goat anti-rabbit secondary antibodies (VC-BA-1000-MM15, Vector Laboratories, Peterborough, UK) and labeled by the avidin-biotin-peroxidase procedure with a commercial immunoperoxidase kit (VECTASTAIN® Elite ABC-Peroxidase Kit Standard, VC-PK-6100-KI01, Vector Laboratories). The immunoreaction was visualized with DAB (Peroxidase DAB Substrate Kit, VC-SK-4100-KI01, Vector Laboratories) substrate, and sections were counterstained with Mayer’s hematoxylin (C0302, Diapath, Italy). Digital image analysis was performed by scoring the number of Ki67-positive cells in three ×400 microscopic fields in the parenchyma of vehicle, IL-4 and CSF-1-treated mice (*n* = 3); monocyte-like cells were excluded from the analysis. For double immune-fluorescence analysis, after heat-induced antigen retrieval in Dewax and HIER BufferH pH 9 (Thermofisher Scientific) and anti-ki67 antibody (RM-9106, Thermofisher Scientific) incubation, goat anti-rabbit green fluorescent antibody (Alexa Fluor 488, Thermo Fisher Scientific) was used; after washing with PBS, sections were re-incubated with anti-GFAP (Z0334, from DAKO Agilent, Santa Clara, CA, USA) or anti-Iba1 (Wako Chemicals USA, Richmond, VA) for 1 h at room temperature followed by a goat anti-rabbit red fluorescent secondary antibody (Alexa Fluor 555, Thermo Fisher scientific). Sections were mounted on coverslips with ProLong Gold Antifade mountant with DAPI (P36941, Thermofisher Scientific).

### Statistical analyses

Unless otherwise stated, all values are expressed as mean ± standard error of the mean (SEM) of *n* observations. The results were analyzed by the Student unpaired two-tailed *t* test using GraphPad Prism 5 software, after a normality test (Kolmogorov-Smirnov) [26]. A value of *p* < 0.05 was considered significant.

## Results

### Peritoneal macrophage proliferation following the local delivery of IL-4

**Fig. 1:**
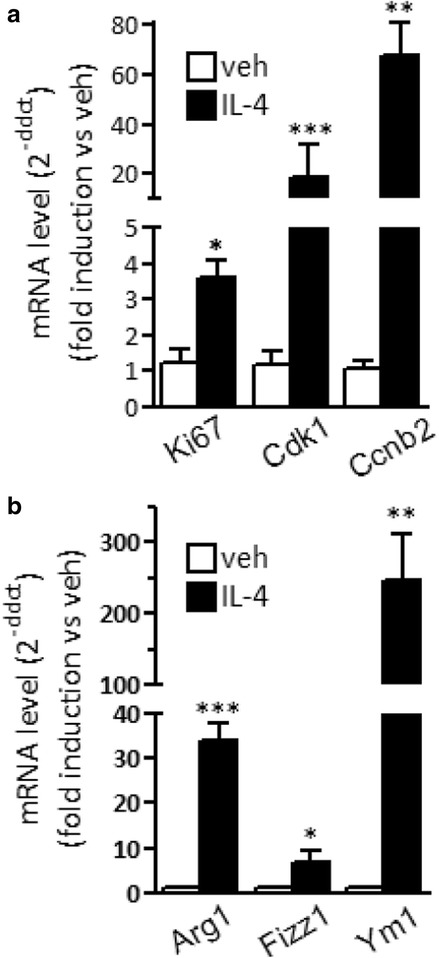
Expression of cell cycle and M2 polarization genes in peritoneal macrophages following IL-4 treatment in vivo. The expression of genes related with **a** cell proliferation (Ki67, Cdk1, and CcnB2) and **b** M2 alternative polarization (Arg1, Fizz1, and Ym1) was analyzed by real-time PCR in peritoneal macrophages isolated from mice treated i.p*.* with vehicle (veh) or 5 μg IL-4 for 24 h. Data sets for each gene were calculated using the 2^-ddCt^ method with respect to the mean value of the vehicle group. Bars represent mean values ± SEM (*n* = 2–6). Student’s unpaired *t* test, *p < 0.05; **p < 0.01; ***p < 0.001 versus veh

In order to analyze the proliferative activity of IL-4 in the brain, we first set up in vivo proliferative assays on macrophages in the peritoneum, a peripheral tissue where resident macrophages were shown to increase in number in response to this immune signal [17]. Peritoneal macrophages were collected from the peritoneal lavage of mice 24 h after an i.p*.* injection of IL-4 and further purified by immunosorting; a control group of mice were injected with vehicle alone. As shown in Fig. 1a, the levels of mRNA coding for proteins related with proliferation (Ki67) and cell cycle (CcnB2 and Cdk1) were increased following IL-4 injection as compared to control mice, suggesting a proliferative effect of IL-4; as expected, the expression of M2 genes, namely Arg1, Fizz1, and Ym1, was also strongly induced by IL-4 (see Fig. 1b).

**Fig. 2:**
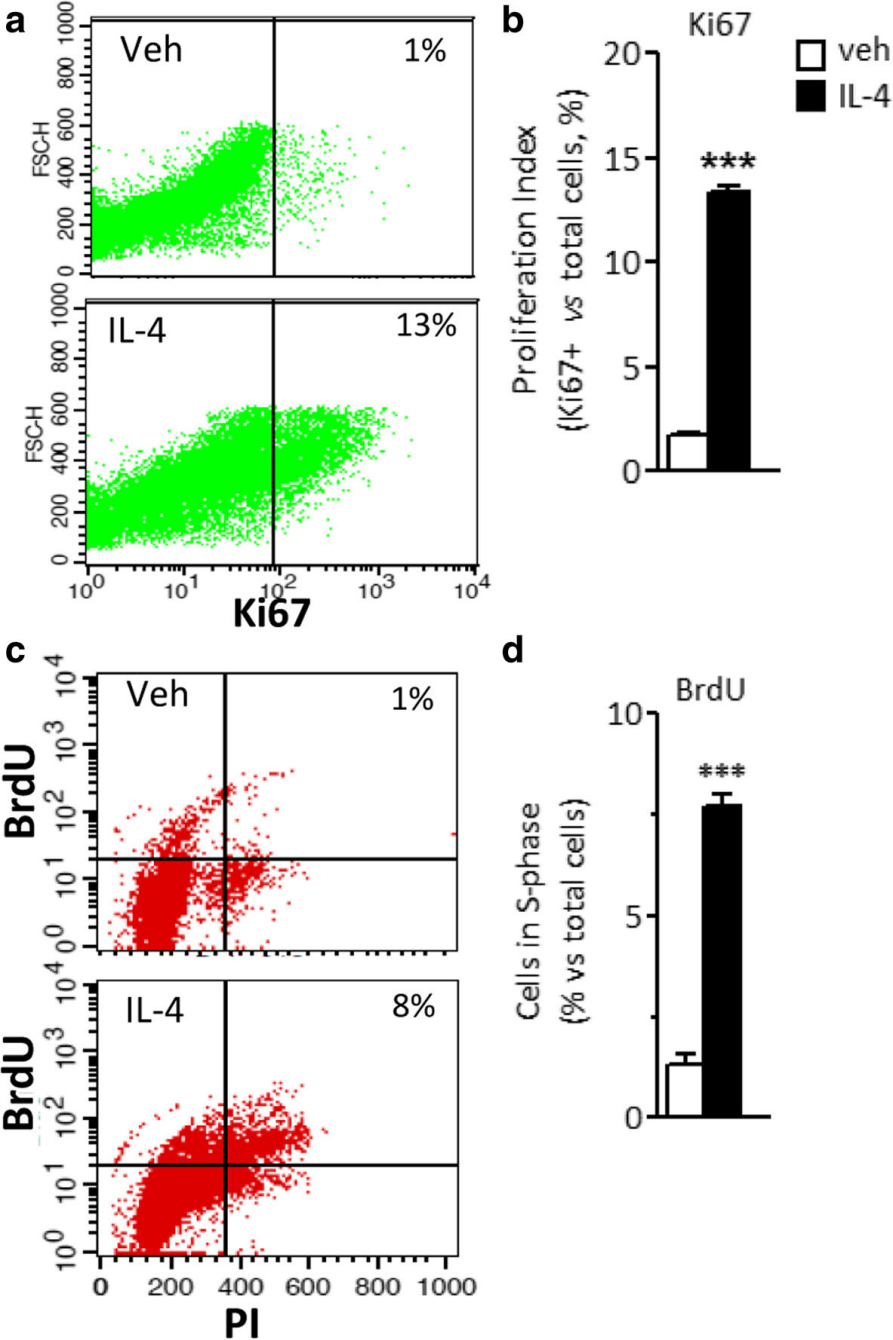
Proliferation of peritoneal macrophages following IL-4 treatment in vivo*.* Ki67 protein expression (**a**, **b**) and BrdU incorporation (**c**, **d**) were analyzed in peritoneal macrophages isolated from mice treated i.p*.* with vehicle (veh) or 5 μg IL-4 for 24 h. **a**, **c** show representative dot plots depicting gating schemes for Ki67 and BrdU analyses, respectively. Bar charts represent the percentage of macrophages showing a positive signal for Ki67 (**b**) or BrdU (**d**) with respect to the total number of macrophages obtained from each mouse following the specified treatment. Bars represent the mean ± SEM of six mice per group. Student’s unpaired *t* test, ***p < 0.001

In order to confirm the effect of IL-4 on cell cycle entry and DNA synthesis, FACS analyses were conducted on peritoneal macrophages to evaluate Ki67 protein levels and BrdU incorporation, respectively. The results shown in Fig. 2 demonstrate that IL-4 is a proliferative signal for peritoneal macrophages, leading to an increase in ki67-positive and in duplicating peritoneal macrophages of 10 and 6%, respectively. Accordingly, the number of recovered macrophages from peritoneal fluid was more elevated in IL-4-treated mice (data not shown).

Altogether, these results show that locally delivered IL-4 is a proliferative signal for peritoneal macrophages.

### Brain delivery of IL-4 has limited effects on brain cell proliferation

**Fig. 3:**
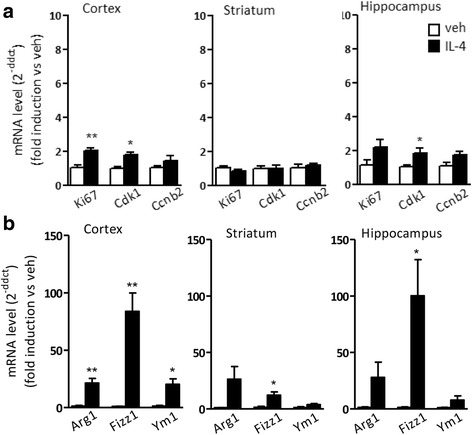
Brain expression of cell cycle and M2 polarization genes following local delivery of IL-4. Mice were injected i.c.v. with vehicle (veh) or 250 ng IL-4; the cortex, striatum, and hippocampus were isolated and analyzed by real-time PCR for the expression of genes related with **a** cell cycle (Ki67, CcnB2, and Cdk1) and **b** M2 alternative polarization (Arg1, Fizz1, and Ym1). Data sets for each gene were calculated using the 2^-ddCt^ method with respect to the mean value of the vehicle group. Bars represent mean values ± SEM (*n* = 5). Student’s unpaired *t* test, *p < 0.05; **p < 0.01 versus veh

We then extended our observation to evaluate the proliferative response of microglia following IL-4 injection in the cerebral ventricles of mice. Cell cycle gene expression was analyzed by real-time PCR in diverse brain areas and, unexpectedly, the mRNA levels coding for Cdk1 and Ki67 were only slightly increased by IL-4, with a twofold induction compared to control samples in the cortex and hippocampus and not in the striatum, while Ccnb2 expression was not modified in any brain areas analyzed (see Fig. 3a). On the other hand, the i.c.v. injection of IL-4 induced the expression of M2 polarization signals, as shown in Fig. 3b: a strong induction of Arg1, Fizz1, and YM-1 mRNAs was generally observed in all brain areas with different region-specific intensities, as already reported [24]. Thus, IL-4-induced polarization is associated with little proliferative effects in the brain.

**Fig. 4:**
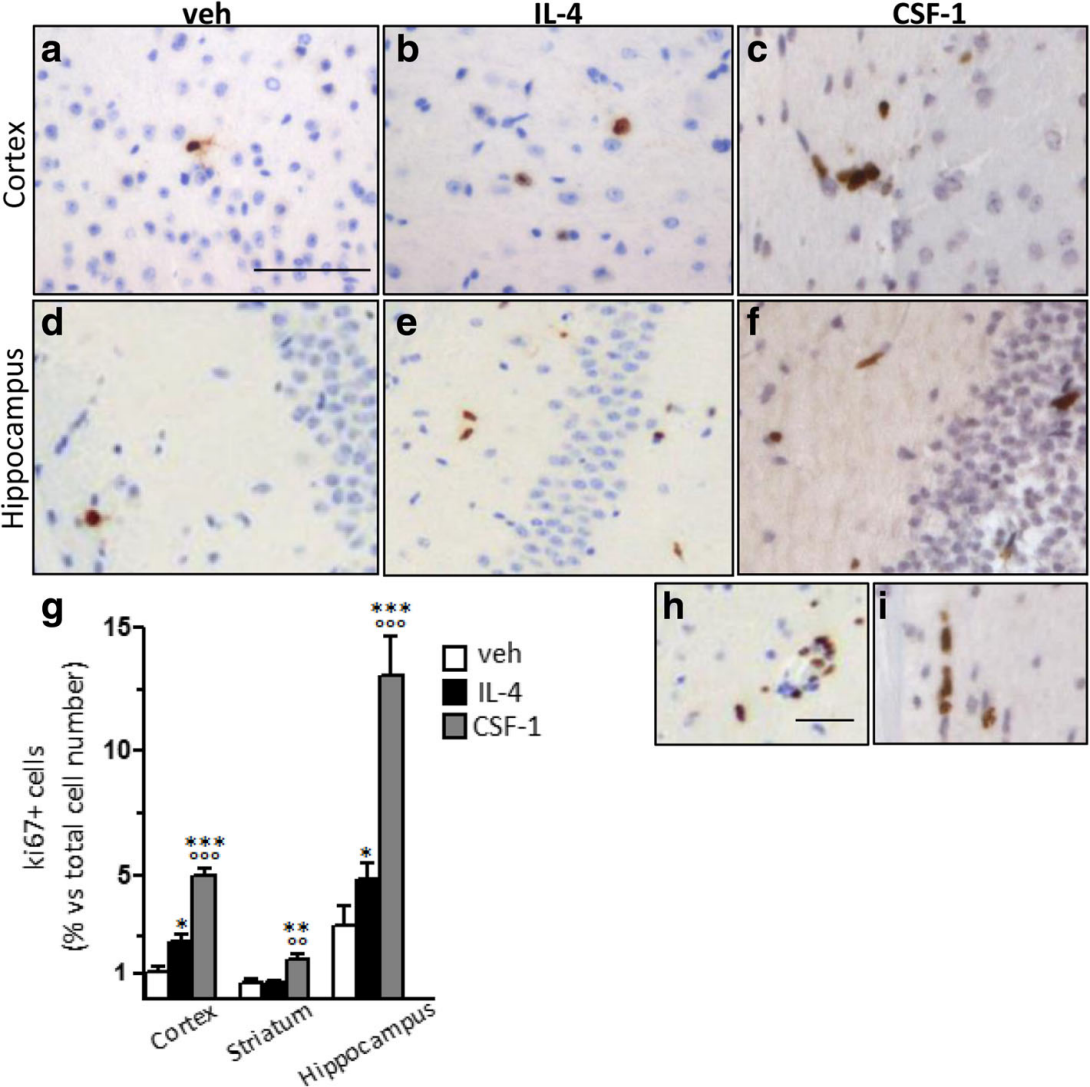
Ki67 protein expression in brain areas following local delivery of IL-4 and CSF-1. Following the i.c.v. injection of vehicle (veh; **a**, **d**), 250 ng IL-4 (**b**, **e**, and **h**) and 1 μg CSF-1 (C, F and J) brains were processed by immunohistochemistry to visualize Ki67 expression. Images were taken from the cortex (**a**–**c**) and hippocampus (**d**–**f**) of mice. Histograms in **g** show the quantification of the percentage of Ki67-positive cells with respect to the total number of cells in brain areas, as specified; **h**, **j** monocyte-like cells were detected in brain vessels from IL-4- and CSF-1-treated mice, respectively and excluded from the counting. Bars represent mean values ± SEM (*n* = 3). Student’s unpaired *t* test, *p < 0.05 versus veh; ° *p* < 0.05 versus IL-4. Scale bar, 100 μm

To further investigate brain cell proliferation following IL-4, we analyzed Ki67 protein expression by immunohistochemistry. The results are reported in Fig. 4 and show that some Ki67-positive cells were present in the cortex and even more in the hippocampus of vehicle-treated mice, while no positively stained cells were detected in the striatum (data not shown); in this experimental conditions, we calculated that Ki67-positive cells are about 1 and 2.7% of total parenchymal cells in the cortex and hippocampus, respectively (see Fig. 4g); IL-4 administration resulted in a statistically significant increase in Ki67-positive cells in the cortex and hippocampus, while no signal was detected in the striatum (see Fig. 4). The morphological appearance of most of the Ki67-positive cells did not recall the microglial cell shape, both in vehicle- and IL-4-treated brains.

It is known that tissue microenvironment substantially shapes macrophage responsiveness. In order to exclude the possibility that general brain-specific mechanisms could silence proliferative signaling pathways in microglia under non-pathological conditions, we evaluated the activity of CSF-1, the best-known proliferative agent for macrophages. Fig. 4c, f shows that the i.c.v delivery of CSF-1 triggers the proliferation of brain cells, some of which displaying a microglia-like morphology, and results in a fivefold increase in ki67-positive cells in the cortex and hippocampus with respect to vehicle and twofold in the striatum, effects that are also significantly different from those obtained with IL-4 (see Fig. 4g). In addition, some Ki67-positive monocyte-like cells were observed in brain vessels after both IL-4 and CSF-1 treatments (see Fig. 4e, j), suggestive of the recruitment of proliferating monocytes.

Altogether, these data show that, in the adult mouse brain, an increase in IL-4 level does not associate with a proliferative response and suggest that this molecule might not be a proliferative signal for microglia, differently from its effects on peritoneal macrophages.

### Proliferative activity of M2 polarization signals on microglia in vivo and in vitro

**Fig. 5:**
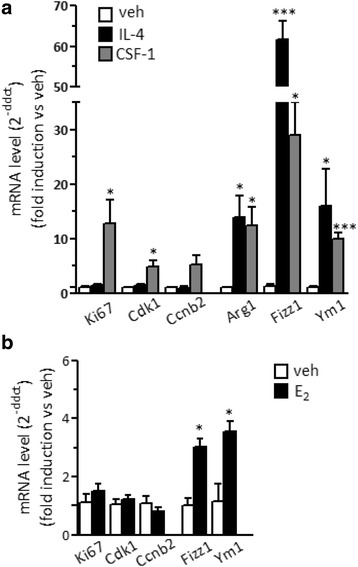
In vivo effects of M2 polarization signals on microglia gene expression. Microglia were isolated by immunosorting 24 h after **a** i.c.v. injections of vehicle (veh; open boxes), 5 μg IL-4 (closed boxes) or 1 μg CSF-1 (gray boxes) or **b** s.c. 17β-estradiol (E_2_) treatment (5 μg/kg). The expression of genes related with active replication and cell cycle (Ki67; Cdk1, Ccnb2) and M2 alternative polarization (Arg1, Fizz1, Ym1) was analyzed by real-time PCR. Data sets for each gene were calculated using the 2^-ddCt^ method with respect to the mean value of the vehicle group. Bars represent mean values ± SEM (*n* = 4). Student’s unpaired *t* test, *p < 0.05; **p < 0.01; ***p < 0.001 versus veh

Since microglia represent only 10% of all brain cells, and considering the restricted percentage of IL-4 responder cells in terms of proliferation in the periphery (see Fig. 2), we evaluated whether the mild proliferative response reported in Fig. 3a could be ascribed to a small subset of microglia. Microglia were purified from the brain of vehicle or IL-4-injected mice and analyzed for proliferative parameters. Notably, Ki67 and cell cycle mRNA levels were not increased in response to i.c.v. IL-4, as shown in Fig. 5a; on the contrary, increased levels of mRNA coding for proteins related with M2 polarization (Arg1, Fizz1, and Ym1) were detected in microglia purified from IL-4 as compared to vehicle-treated mice. These results suggest that brain cells other than microglia are induced to moderately proliferate in response to IL-4.

On the contrary, CSF-1 brain delivery resulted in a significantly increased expression of genes related with proliferation and cell cycle specifically in microglia, as shown in Fig. 5a; as expected since CSF-1 is endowed with M2 polarization effects, we observed a parallel enhancement of M2 polarization genes such as Arg1, Fizz 1, and Ym1.

We then asked whether 17-beta-estradiol (E_2_), a lipophilic hormone that reduces microglia inflammatory phenotype, could alter microglia proliferation in line with the recent observation on its proliferative effect on peripheral macrophages [22, 23, 27, 28]. Similar to what is observed with IL-4, systemic administration of physiological doses of E_2_ caused a significant increase in the mRNA coding for Fizz1 and Ym1 in microglia, while cell cycle genes were unaffected (Fig. 5b).

Thus, these data show that under homeostatic conditions, microglia can proliferate in response to some M2-activating signals, such as CSF-1, but not IL-4 and E_2_.

**Fig. 6:**
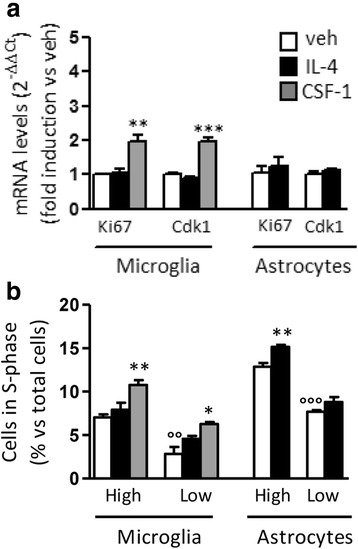
Proliferative responses of primary cultures of microglia and astrocytes. **a** The expression of proliferation genes (Ki67 and Cdk1) was analyzed in primary cultures of microglia and astrocytes following vehicle (veh; open boxes), 20 ng/ml IL-4 (closed boxes) or 20 ng/ml CSF-1 (gray boxes) treatments. Bars represent the mean ± SEM of three independent experiments, each performed in triplicate. Data sets for each gene were calculated using the 2^-ddCt^ method with respect to the mean value of the vehicle group. Bars represent mean values ± SEM (*n* = 3). Student’s unpaired *t* test, **p < 0.01; ***p < 0.001. **b** BrdU incorporation was evaluated by flow cytometry in microglia and astrocytes treated with 20 ng/ml IL-4 (closed boxes) or 20 ng/ml CSF-1 (gray boxes) added to culture medium containing high (15%) or low (5%) serum concentrations. Bars represent the mean ± SEM of three independent experiments, each performed in triplicate (*n* = 3). Student’s unpaired *t* test, *p < 0.05; **p < 0.01; ***p < 0.001 versus vehicle; °°°*p* < 0.001 versus vehicle high FBS

During the perinatal period, microglia proliferation and development are strongly active processes; we thus asked whether IL-4 might associate with proliferative effects on microglia from this developmental stage. As shown in Fig. 6a, IL-4 treatment of microglia primary cultures from newborn animals did not modify Ki67 and Cdk1 expression, while CSF-1 still resulted in increased Ki67 and Cdk1 mRNA levels. The ability of microglia to proliferate in response to CSF-1 was further demonstrated by BrdU incorporation analyses as a biological evidence of proliferation, while no signs of proliferation were observed following IL-4 under different experimental conditions such as reduced serum concentrations (see Fig. 6b). Gene expression analyses demonstrated that the alternative phenotype is induced in these cells by IL-4 and CSF-1, although with different intensities (see Additional file 2: Figure S1A). Lack of a proliferative response of microglia could not be ascribed to a reduced sensibility of microglia, since the expression of the IL-4 receptor (IL-4Rα) in ex vivo-sorted or in vitro microglia did not vary in response to IL-4 (see Additional file 2: Figure S1B). We also tried to extend our observation to other primary macrophages obtained from adult animals; however, CSF-1 as well as IL-4 and E_2_ failed to induce proliferation of peritoneal macrophages while still retaining their polarization effects (see Additional file 3: Figure S2), in agreement with recent observation using similar experimental conditions [22, 29].

In parallel, primary cultures of astrocytes were also assayed for their proliferative response to IL-4; although cell cycle gene expression was unchanged, BrdU incorporation assay revealed a slight yet significant increase in the number of astrocytes in the S-phase of the cell cycle following IL-4, suggesting that the proliferative response observed in the brain following i.c.v. IL-4 (reported in Figs. 3 and 4) can be ascribed, at least in part, to astrocytes. Indeed, double immune staining of brain sections following i.c.v IL-4 shows co-localization of Ki67 signal with about 1% of GFAP-positive cells (see Additional file 4: Figure S3), and not Iba1-positive cells.

**Fig. 7:**
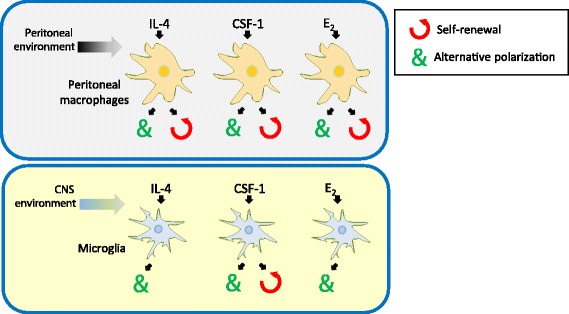
Tissue-specific responses of resident macrophages to polarization signals. Resident macrophages in the peritoneum show proliferative and alternative polarization responses to IL-4, CSF-1, and E_2_; in the brain, alternative polarization of microglia is induced by the same signals, although with different intensities and target gene-specificity, while cell proliferation is restricted to CSF-1 signaling

Altogether, these data show that microglia in the brain of adult mice are activated by M2 signals, such as IL-4, CSF-1, and E_2_, to shape their immune phenotype while self-renewal is specifically triggered by CSF-1; IL-4 and E_2_ brain activities do not correlate with microglia cell expansion, in contrast to what has been observed for peripheral macrophages, as summarized in Fig. 7.

## Discussion

The present study provides the first evidence to show that microglial cells do not proliferate in response to IL-4, at least in the present experimental conditions; in fact, using molecular and biological assays, we were unable to detect microglia proliferation following IL-4 treatment both in vivo in the mouse brain and in vitro on primary cultures microglia from newborn animals. On the contrary, this immune mediator enhances the proliferation of peripheral macrophages, as shown in this and previous studies [17]. Moreover, also another M2-activating signal, namely E2, showed a similar polarization and non-proliferative activity in microglia of adult mice.

Microglial population is expanded during the pathological course of neurodegenerative diseases and contributes to disease progression [7–12]; the activity of IL-4 in brain recently received considerable attention in relation with its anti-inflammatory and pro-resolution effects that are directly mediated by microglia. Expression and secretion of IL-4 is induced by neuroinflammatory insults in activated immune cells and damaged neurons and IL-4-activated microglia produce a series of mediators, including neurotrophic factors, matrix remodeling, and proteolytic enzymes that help reduce neuroinflammation and promote tissue repair, as shown in experimental models of neurodegenerative diseases [18, 21, 30–32]. It is thus important to understand whether IL-4 contributes to the expansion of microglia that undergoes an alternative activation phenotype. In this scenario, our results provide an important step forward, as we show that IL-4 activity in the brain, although associated with immunoregulatory and tissue repair phenotype, does not correlate with an increase in microglia number. Analogously, estrogen action in microglia has been proposed to mediate the protective effects observed for this hormone against neurodegenerative diseases in humans and animal models, while the reduction in estrogen levels that occurs at menopause is associated with an increased incidence of neuroinflammatory pathologies [33]. Previous studies reported the effects on microglia polarization when E_2_ was assayed together with neuro-immune signals [23, 34]; the present study is the first evidence to show that E_2_ per se promotes microglia alternative polarization.

Our results highlight a substantial difference between microglia and peripheral macrophage biology in their responsiveness to M2-activating signals, as shown in Fig. 7. These two resident macrophage populations have a different developmental origin and acquire both common and specialized functions during life; indeed, a marked difference in the gene expression profiles has been observed in microglia as compared with other macrophage populations [35]. Moreover, it is known that the surrounding microenvironment is crucial to modulate macrophage functions by providing tissue-specific signals and interactions that influence cell phenotype and responsiveness. However, explanations for the lack of microglia proliferation are still missing; one could hypothesize that specific alterations of the IL-4 signaling pathway that converges on cell proliferation are present in microglia as compared to peripheral macrophages; a possible candidate is the phosphatidylinositol-3 kinase (PI3K)/Akt pathway, which has been shown to be necessary for the IL-4 activity on the proliferative burst of peripheral macrophages [29, 36]; instead, proliferative responses of macrophages to E_2_ are still ill defined. On the other hand, our data show that the proliferative signaling of CSF-1R is maintained in microglia, in agreement with previous reports [7, 37–39] which also associated CSF-1 activity with the PI3K pathway. Thus, the specific effects of CSF-1 and IL-4 signaling pathways on microglia proliferation might either involve cell-specific mediators of the PI3K pathway or yet undefined PI3K-unrelated processes.

Our study also shows that the in vivo effects of CSF-1 extend to the induction of an M2-like phenotypic activation of brain cells, similar to the response of peritoneal macrophages in vivo, as shown here, or in vitro, as previously reported [40]; interestingly, an opposite effect on Fizz1 expression in brain and peritoneal macrophages was observed, as well as the induction of specific M2 genes in primary cultures of microglia; these features need future investigation, although the experimental procedures and development stage used for culturing primary microglia cells may alter cell responsiveness and limit any comparison with resident microglia of the adult brain. The use of CSF-1R inhibitors in experimental models of neurodegenerative pathologies has been shown to reduce microglia density and to associate with beneficial effects against disease progression, although still unclear are the consequences of CSF-1R inhibition on polarized phenotype effectors, as well as the role of CSF-1R-independent and/or compensating pathways in microglia self-renewal and turnover [5, 7, 11, 41, 42]. Environmental and pathological signals seem to influence the type, timing, and resolution of microglia polarization responses through ill-defined mechanisms that differ between the healthy and injured brain and that need deeper investigation.

## Conclusions

The uncoupling of the polarization and proliferative activities of IL-4 and E_2_ in microglia is unclear; indeed, several aspects of microglia polarization have not been yet investigated, such as the fate of these cells following activation or the existence of local amplifiers of alternative polarization responses, which have only recently been identified for IL-4 in some peripheral tissues [43]. Thus, further studies are needed to understand the molecular mechanisms of microglia proliferation, with the aim of opening novel therapeutic strategies that selectively and timely potentiate alternatively activated microglia while avoiding inflammatory macrophage expansion and monocyte recruitment.

## Additional files

Additional file 1: Table S1. Oligonucleotides used in real time PCR assays. (PDF 182 kb)

Additional file 2: Figure S1. In vitro polarization responses of microglia to IL-4 and CSF-1. A) The expression of M2 polarization genes (Arg1 and Mrc1) was analyzed in primary cultures of microglia following the treatment with vehicle (veh; open boxes), 20 ng/ml IL-4 (closed boxes) or 20 ng/ml CSF-1 (gray boxes). B) The expression of IL-4Rα was analyzed either in microglia cells obtained by immunosorting from the brain of vehicle or IL-4 icv-injected mice (ex vivo) or in primary cultures of microglia (in vitro) as specified above. Bars represent the mean ± SEM of 3 independent experiments, each performed in triplicate. Data sets for each gene were calculated using the 2^-ddCt^ method with respect to the mean value of the vehicle group. Bars represent mean values ± SEM (*n* = 3). Student’s unpaired *t*-test, *p < 0.05; **p < 0.01; ***p < 0.001. (PDF 167 kb)

Additional file 3: Figure S2. In vitro proliferative and polarization responses of macrophages to IL-4, CSF-1 and E_2_. A) The expression of proliferation (Ki67, Cdk1 and Ccnb2) and M2 polarization (Arg1, Fizz1, Mrc1) genes was analyzed in primary cultures of peritoneal macrophages following treatment with 20 ng/ml IL-4 (black bars) or 20 ng/ml CSF-1 (gray bars). B) The expression of proliferation (Ki67, Cdk1 and Ccnb2) and M2 polarization (Arg1, Vegfα) genes was analyzed in primary cultures of peritoneal macrophages following vehicle (0.01% EtOH) or 10^−5^ M E_2_ treatments. Bars represent the mean ± SEM of 3 independent experiments, each performed in triplicate Data sets for each gene were calculated using the 2^-ddCt^ method with respect to the mean value of the vehicle group. Bars represent mean values ± SEM (n = 3). Student’s unpaired *t*-test, *p < 0.05; ***p < 0.001. (PDF 339 kb)

Additional file 4: Figure S3. Cellular localization of Ki67 immunostaining in brain astrocytes. Brain sections were analyzed by immunohistochemistry for the expression of Ki67 using antibodies against Ki67 (green labeling) and GFAP (red staining) following the icv administration of 250 ng IL-4. Ki67-positive cells co-localize with GFAP-positive cells (white arrows). Scale bar, 10 μm. (PDF 105 kb)

## Abbreviations

BSA: Bovine serum albumin
CNS: Central nervous system
CSF-1: Colony-stimulating factor-1
DAB: 3,3′-diaminobenzidine
E_2_: 17β-estradiol
FBS: Fetal bovine serum
HBSS: Hank’s balanced salt solution
i.c.v*.*: Intracerebroventricular
IL-4: Interleukin-4
MEM: Minimum essential Eagle’s medium
PBS: Phosphate-buffered saline
PCR: Polymerase chain reaction
SEM: Standard error of the mean
