# Supplementary material for: Selective proliferative response of microglia to alternative polarization signals
Source: J Neuroinflammation. 2017 Dec 4;14:236. doi: 10.1186/s12974-017-1011-6 (PMC5715534; doi:10.1186/s12974-017-1011-6)
Supplement: Supplementary file 1 — Oligonucleotides used in real time PCR assays. (PDF 182 kb) [file 12974_2017_1011_MOESM1_ESM.pdf]

Supplementary table 1. Oligonucleotides used in real-time PCR assays

Gene	Forward sequence	Reverse sequence
mKi67	5'-AGAGCTAACTTGCGCTGACT-3'	5'-TCAATACTCCTTCCAAACAGGCA-3'
mCdk1	5'-ACACGAGGTAGTGACGCTGT-3'	5'-TCAATCTCTGAGTCGCCGTG-3'
mCcnB2	5'-CCGACGGTGTCCAGTGATTT-3'	5'-CTGAGGTTTCTTCGCCACCT-3'
mArg1	5'-GAATCTGCATGGGCAACCT-3'	5'-ACACGATGTCTTTGGCAGATAT-3'
mFizz1	5'-GGAACTTCTTGCCAATCCAGC-3'	5'-AAGCCACAAGCACACCCAGT-3'
mYm1	5′-GAAGGAGCCACTGAGGTCTG-3′	5′-GAGCCACTGAGCCTTCAAC-3′
mMrc1	5'-TTCAGCTATTGGACGCGAGG-3'	5'-GAATCTGACACCCAGCGGAA-3'
mVegfα	5'-AGCAGAAGTCCCATGAAGTGA-3'	5'-ATGTCCACCAGGGTCTCAAT-3'
rKi67	5'-CCACAACCAGGAAGACCAGTT-3'	5'-TGATCCCATTATCCGCCTGC-3'
rCdk1	5'-GGGAACAGAGAGGGTCCGTT-3'	5'-ATTTCCCGGATTGCCGTACT-3'
rArg1	5'-ACAAGACAGGGCTACTTTCAGG-3'	5'-ACAAGACAAGGTCAACGCCA-3'
rMrc1	5'-CAACTCTTGGACTCACGGCA-3'	5'-ATGATCTGCGACTCCGACAC-3'
mIL-4Ra	5'-AACTCGCAGGTTCTGGCTGG-3'	5'-AAGCCCCGAGTCCTAGGTT-3'
36B4	5′-GGCGACCTGGAAGTCCAACT-3	5′-CCATCAGCACCACGGCCTTC-3′

m = mouse; r = rat
