# Supplementary figures and images for: Selective proliferative response of microglia to alternative polarization signals
Source: J Neuroinflammation. 2017 Dec 4;14:236. doi: 10.1186/s12974-017-1011-6 (PMC5715534; doi:10.1186/s12974-017-1011-6)

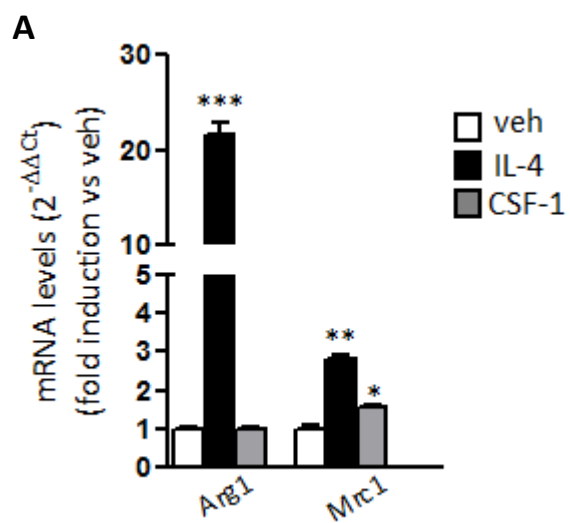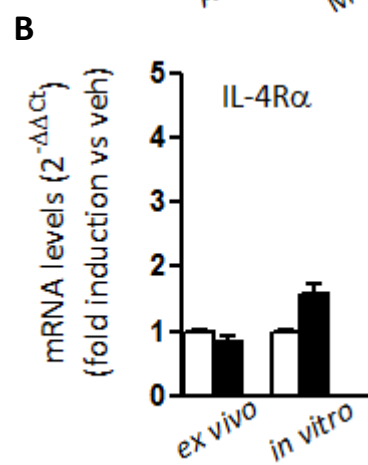

Supplementary Figure 1.

Supplement: Supplementary file 2 — In vitro polarization responses of microglia to IL-4 and CSF-1. A) The expression of M2 polarization genes (Arg1 and Mrc1) was analyzed in primary cultures of microglia following the treatment with vehicle (veh; open boxes), 20 ng/ml IL-4 (closed boxes) or 20 ng/ml CSF-1 (gray boxes). B) The expression of IL-4Rα was analyzed either in microglia cells obtained by immunosorting from the brain of vehicle or IL-4 icv-injected mice (ex vivo) or in primary cultures of microglia (in vitro) as specified above. Bars represent the mean ± SEM of 3 independent experiments, each performed in triplicate. Data sets for each gene were calculated using the 2-ddCt method with respect to the mean value of the vehicle group. Bars represent mean values ± SEM (n = 3). Student’s unpaired t-test, *p < 0.05; **p < 0.01; ***p < 0.001. (PDF 167 kb) [file 12974_2017_1011_MOESM2_ESM.pdf]

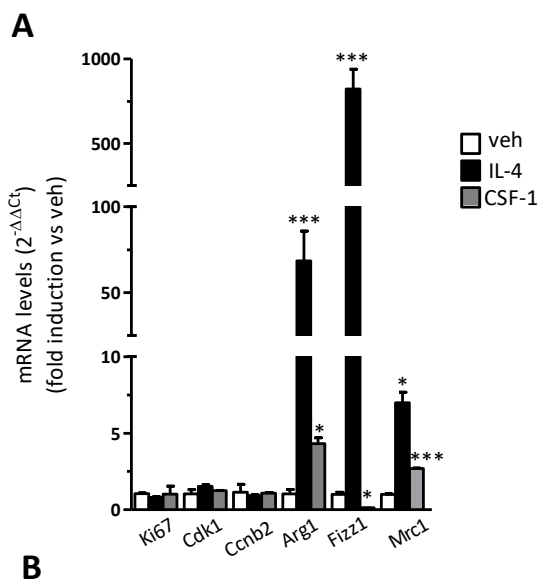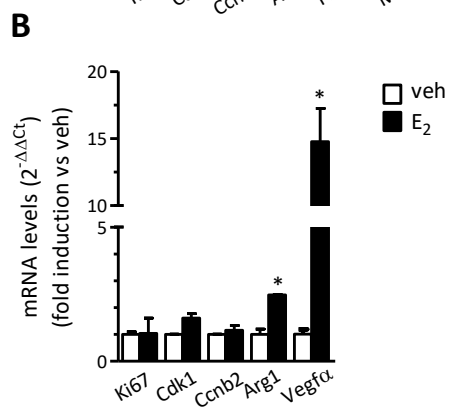

**Supplementary Figure 2.**

Supplement: Supplementary file 3 — In vitro proliferative and polarization responses of macrophages to IL-4, CSF-1 and E2. A) The expression of proliferation (Ki67, Cdk1 and Ccnb2) and M2 polarization (Arg1, Fizz1, Mrc1) genes was analyzed in primary cultures of peritoneal macrophages following treatment with 20 ng/ml IL-4 (black bars) or 20 ng/ml CSF-1 (gray bars). B) The expression of proliferation (Ki67, Cdk1 and Ccnb2) and M2 polarization (Arg1, Vegfα) genes was analyzed in primary cultures of peritoneal macrophages following vehicle (0.01% EtOH) or 10−5 M E2 treatments. Bars represent the mean ± SEM of 3 independent experiments, each performed in triplicate Data sets for each gene were calculated using the 2-ddCt method with respect to the mean value of the vehicle group. Bars represent mean values ± SEM (n = 3). Student’s unpaired t-test, *p < 0.05; ***p < 0.001. (PDF 339 kb) [file 12974_2017_1011_MOESM3_ESM.pdf]

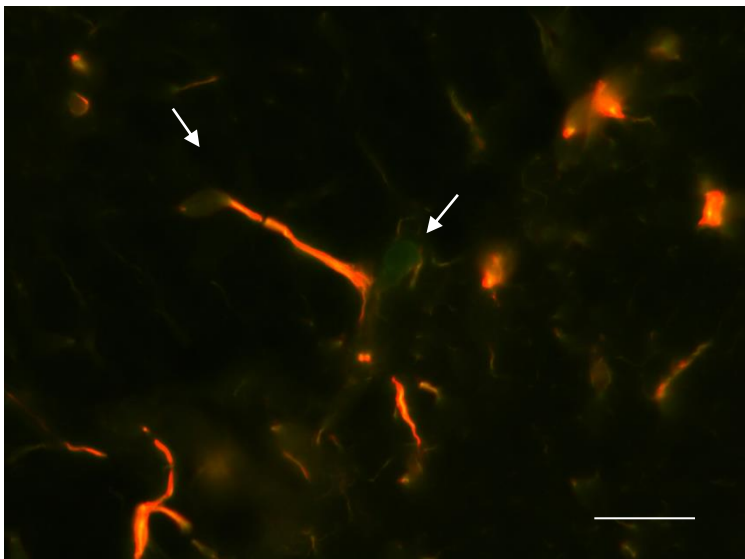

Supplementary Figure 3

Supplement: Supplementary file 4 — Cellular localization of Ki67 immunostaining in brain astrocytes. Brain sections were analyzed by immunohistochemistry for the expression of Ki67 using antibodies against Ki67 (green labeling) and GFAP (red staining) following the icv administration of 250 ng IL-4. Ki67-positive cells co-localize with GFAP-positive cells (white arrows). Scale bar, 10 μm. (PDF 105 kb) [file 12974_2017_1011_MOESM4_ESM.pdf]
